# Two rare genitourinary tuberculosis presentations with isolated testicular and tubo-ovarian tuberculosis in resource limiting setups: A case report and review of literature

**DOI:** 10.1016/j.ijscr.2025.111026

**Published:** 2025-02-09

**Authors:** Wondale Tsega Tebeje, Addisu Assfaw Ayen, Dagne Aschenaki Argaw, Tadie Siraw Mulu, Abdirahman Ahmed Abdulahi, Wali Ahmed Nur

**Affiliations:** a Department of Internal Medicine, Debre Tabor University, Debre Tabor, Ethiopia; b Department of general surgery, Gerbo primary hospital, Somalia, Ethiopia; c Department of obstetrics and gynecology, Gerbo primary hospital, Somalia, Ethiopia; d General practitioner, Gerbo primary hospital, Somalia, Ethiopia; e Bachelor degree radiology technology, Masters on public health, Gerbo primary hospital, Somalia, Ethiopia

**Keywords:** Genitourinary TB, Isolated testicular TB, Tubo-ovarian abscess, Case report, Somalia, Ethiopia

## Abstract

**Introduction and importance:**

Tuberculosis (TB), a common chronic infectious disease, affected approximately 10.6 million people worldwide in 2021. While TB can affect the lungs (pulmonary TB) or other parts of the body, extrapulmonary TB, genital TB is a rare form of extrapulmonary TB. Within genital TB, isolated testicular TB is uncommon; representing only 2–4 % of genitourinary TB cases, and ovarian TB is also infrequent in females.

**Case presentation:**

Two patients from the Gerbo region of Somalia and Ethiopia presented with: a 65-year-old man with a 10-month history of left scrotal swelling and mild pain, and a 40-year-old multiparous pregnant woman with a 5-day history of acute abdominal pain and distension resulting in abortion. Neither patient reported chronic comorbidities or cough. The male patient was stable with normal initial investigations, while the female patient presented with fever and ascites. Diagnosis was confirmed by histopathological examination and AFB staining in the male patient and a positive GeneXpert test on intra-abdominal fluid in the female patient. Both patients received anti-tuberculosis treatment (2RHZE/4RH), resulting in complete recovery.

**Clinical discussion:**

Despite global efforts, tuberculosis remains a leading cause of death worldwide, disproportionately impacting low-socioeconomic populations. Genitourinary tuberculosis (GUTB), representing 30–40 % of extrapulmonary TB cases (second only to lymph node involvement), commonly affects the kidneys and fallopian tubes. Isolated testicular and tubo-ovarian tuberculosis are rare forms of GUTB. Immunocompromise increases the risk of genital TB, although neither of our patients exhibited such conditions. Genital TB presents variably depending on sex and affected organs. Diagnosis relies on isolating Mycobacterium from various samples and histological findings. Guideline-directed anti-tuberculosis therapy typically cures the infection without surgery.

**Conclusion:**

Genital tuberculosis requires a high index of suspicion for accurate diagnosis, particularly in resource-limited settings. Effective anti-tuberculosis treatment often suffices, minimizing the need for surgery.

## Highlights

• Isolated testicular tuberculosis is a rare form of genitourinary TB.
• This rare extrapulmonary TB manifestation can present acutely, potentially causing abortion.
• The clinical presentation can mimic tumors, posing a diagnostic challenge.
• A high index of suspicion for tuberculosis is crucial, necessitating thorough investigation before surgical intervention is considered

## 1 Introduction

Worldwide in 2021 around 10.6 million of individuals were affected with tuberculosis (TB) [1]. TB is a common chronic infectious disease, presents with a variety of manifestations, affecting either the lungs (pulmonary) or other parts of the body (extrapulmonary) [2]. Genitourinary tuberculosis(GUTB) is the second most common form of extrapulmonary TB, after lymph node involvement. Isolated testicular tuberculosis is rare, accounting for only 2–4 % of genitourinary TB cases [3]; while ovarian tuberculosis accounts for approximately one-quarter of all cases of genital tuberculosis, which itself represents about 1 % of all tuberculosis cases [4]. Testicular TB which can be presented with testicular swelling and can be confused with testicular tumor and other infectious processes [5]; while tubo-ovarian tuberculosis can present with a wide range of symptoms, mimicking those of an ovarian tumor, making diagnosis challenging and often requiring sophisticated imaging and laboratory tests to differentiate between the two. The non-specific nature of the symptoms contributes to diagnostic difficulties [6]. In this case report we report 65 years old Ethiopian men who presented with chronic scrotal swelling which confused with testicular tumor and a 35 years old female pregnant mother presented with acute abdominal pain, ascites and abortion.

The case report narrated with Surgical Case Report (SCARE) 2023 guideline [7].

## 2 Case presentation

### 2.1 Case one

**Fig. 1:**
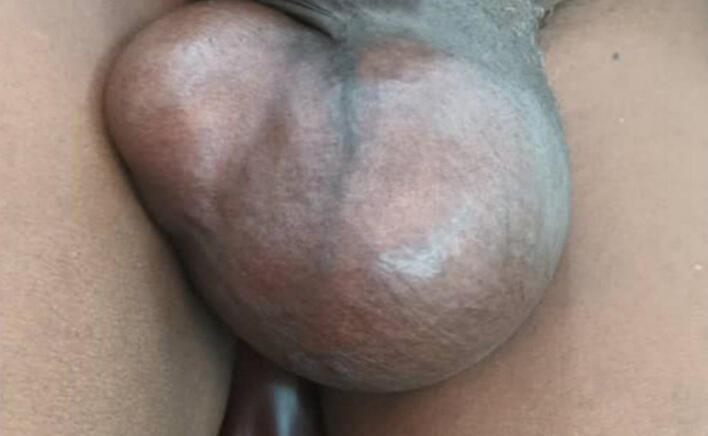
around 4 cm by 6 cm firm, slightly tender left testicular mass.

A 65-year-old man from the Gerbo region of Somalia, Ethiopia, presented with a 10-month history of scrotal swelling. The swelling began on the left side progressively enlarging over five months. He reported mild pain but denied skin discoloration or discharge. A patient presented with increased urinary frequency but no other lower urinary tract symptoms (dysuria, urgency, or hematuria). He denied any comorbid conditions, cough, fever, or night sweats. Physical examination revealed a conscious, stable patient appearing chronically ill but without cardiorespiratory distress. Chest examination was clear and resonant. Genitourinary examination showed a 4 × 6 cm scrotal swelling (Fig. 1) with a smooth surface, firm consistency, irregular borders, and no tenderness, palpable lymphadenopathy, or discharge.

Following an initial impression of a testicular tumor, the patient underwent investigations which included a complete blood count (CBC), organ function tests, serum electrolytes, urinalysis, chest X-ray, fasting blood glucose, and an abdominopelvic ultrasound, all of which were normal. HIV serology and serum VDRL were negative. On scrotal ultrasound revealed left enlarged testes and epididymis with heterogeneous echo texture. Multiple calcifications were present in left testes with normal right one, along with irregular borders and thickened scrotal walls. Based on the suspected testicular tumor, the patient was referred to a tertiary care center where he underwent surgergical biopsy.Following surgery, histopathological examination of the sample revealed granulomatous inflammation with caseous necrosis and acid-fast bacilli (AFB). A diagnosis of testicular tuberculosis was made. Treatment with anti-tuberculosis medication (2RHZE/4RH regimen with pyridoxine), following national and WHO guidelines, was initiated. At one month, wound showed healing, and the patient showed complete resolution upon completion of the anti-tuberculosis therapy.

### 2.2 Case two

A 40-year-old grand multiparous woman from Gerbo, Somali region, Ethiopia, presented with a five-day history of crampy abdominal pain and distension. She also reported vomiting, anorexia, and low-grade intermittent fever. She had been amenorrheic for six months. The patient denied any history of cough, edema, rash, or joint pain, and reported no known medical problems. On examination, the patient's pulse was 134 bpm and temperature was 38.3 °C. Her abdomen was distended, consistent with a 24-week gravid uterus, and showed signs of fluid accumulation. Suprapubic tenderness was noted. Chest examination and other findings were unremarkable.

On investigations; Complete blood count (CBC) showed leukocytosis of 13,000 cells/μL with 60 % neutrophils and 35 % lymphocytes, hemoglobin of 10 g/dL, and a normal platelet count. The erythrocyte sedimentation rate (ESR) was elevated at 60 mm/h. Renal function tests, liver function tests, serum electrolytes, chest X-ray, and blood glucose were within normal limits. Testing for HIV, VDRL, HBsAg, and HCV antibodies was negative. Abdominopelvic ultrasound revealed a 16.2 × 8.9 cm thick-walled, irregular, heterogeneous mass with septations and internal debris involving the right ovary, fallopian tube, and peritoneum, consistent with a tubo-ovarian abscess with ascites. Obstetric ultrasound revealed a singleton intrauterine pregnancy at 24 weeks and 3 days gestational age, with adequate amniotic fluid and no apparent gross congenital anomalies.

The patient was admitted and started on empiric intravenous ceftriaxone and metronidazole. On the fifth day, she experienced an uncomplicated expulsion of a 600-g female abortus. Subsequently, suprapubic pain and abdominal distension increased. Ultrasound-guided aspiration of the abdominopelvic collection was performed. Analysis revealed a total WBC count of 1450 cells with 70 % lymphocytes. The sample was negative for acid-fast bacilli (AFB) and Gram stain, but positive for Xpert MTB/RIF (with no rifampicin resistance detected).

The patient began anti-tuberculosis treatment (2RHZE/4RH regimen) according to national and WHO guidelines, along with supportive care; antibiotics were discontinued. She showed significant improvement, with decreased pain and abdominal distension. A follow-up abdominopelvic ultrasound at one month showed a markedly reduced abdominal collection, with minimal fluid remaining near the right ovary and otherwise normal viscera. At two months, the ultrasound was normal, with no complaints or adverse effects from the anti-tuberculosis medication. Upon completion of treatment, the patient was stable, in good health, with a normal abdominal ultrasound, and discharged from follow-up after receiving family planning counseling.

## 3 Discussion

Despite global efforts, tuberculosis remains among the ten leading causes of death worldwide, disproportionately affecting low-socioeconomic countries [8]. Genitourinary tuberculosis (GUTB) is a common extrapulmonary TB manifestation, comprising 30–40 % of cases, second only to lymph node involvement; which mostly involve kidney and fallopian tube but as our patients presented with isolated testicular involvement and tubo ovarian tuberculosis are a rare form GUTB [9].

Several factors increase the risk of genitourinary tuberculosis (GUTB), including retroviral infection, kidney transplantation, diabetes mellitus, dialysis, and chronic immunosuppressive therapies [10]. However, the patients in this case did not present with any of these identified risk factors. Hematogenous spread is the most common route of tuberculosis dissemination. Although the patients in this instance lacked other identifiable primary foci, direct ascending or descending spread, or spread from contiguous gastrointestinal (GI) structures, peritoneum, or mesenteric lymph nodes remain possibilities [11,12]. Given the second patient's ascites and tubo-ovarian abscess, and the absence of other identifiable infection sources in both patients, spread from a contiguous gastrointestinal source is a plausible explanation for second patient. In males, genital tuberculosis can spread via hematogenous and lymphatic routes, and may also originate from the urinary tract (prostate, epididymis, and seminal vesicles), though this patient showed no evidence of prostate involvement [13].

The clinical presentation of genital tuberculosis (GTB) varies between males and females. While female patients may present with primary or secondary infertility [14] unlike the current multiparous patient with no prior history of abortion, other presentations include fever, pelvic pain with or without ascites (potentially mimicking ovarian tumors) as our patient presented with ovarian involvement with ascites while others they can present with menstrual irregularities, or vaginal discharge [15,16]. Approximately 80 % of patients present with genital tuberculosis (TB) between the ages of 20 and 40 years; the patient's age of 40 falls within the upper range of this demographic [17]. Other systemic manifestations, such as anorexia, weight loss, and night sweats, can also occur in female genital TB [18].

In contrast to the female presentation, males with genital tuberculosis may present with a variety of symptoms. Approximately two-thirds experience unilateral scrotal swelling, with or without pain (similar to the current patient), while the remaining one-third present with bilateral involvement [19]. Other manifestations include lower urinary tract symptoms such as frequency, urgency, and dysuria, although in this case, only frequency was noted [20]. Infertility may also occur as a complication, but most patients lack systemic symptoms like fever, weight loss, and anorexia, consistent with the presentation of this patient [21].

Diagnosis of genital tuberculosis (TB) in both sexes relies on isolating Mycobacteria via methods such as GeneXpert, culture, or acid-fast bacilli (AFB) staining of samples like urine, serous fluid, or discharge. Histopathological examination of fine-needle aspirates (FNAC) or surgical biopsies may also reveal granulomatous inflammation with caseous necrosis and the presence of AFB, as seen in the first male case. Alternatively, as in the second male case, Mycobacteria may be identified by GeneXpert testing of intra-abdominal fluid [20].

Genital tuberculosis is managed similarly to pulmonary tuberculosis, typically using a 2RHZE/4RH anti-tuberculosis regimen. Surgical intervention is usually not necessary, and our both patient's improvement without surgery supports this approach.

## 4 Conclusion

Genital tuberculosis is a rare extrapulmonary manifestation, posing diagnostic challenges, particularly in resource-limited settings where it may be confused with other pathologies like tumors. A high index of suspicion is crucial, and appropriate investigations should be performed before surgical intervention is considered. In many cases, effective anti-tuberculosis treatment alone results in a positive outcome, obviating the need for surgery.

## Abbreviations

AFB: Acid-fast bacillus
GUTB: Genitourinary tuberculosis
PLT: Platelet
TB: Tuberculosis
2RHZE: Isoniazid, Rifampin, Ethambutol, and Pyrazinamide for 2 months
4RH: Rifampicin and Isoniazid for 4 months
WBC: white blood cell

## Consent

Written informed consent was obtained from the patient for publication and any accompanying images. A copy of the written consent is available for review by the Editor-in-Chief of this journal on reques**t.**

## Declaration of Generative AI and AI-assisted technologies in the writing process

AI language modelling tools were utilized for the improvement of English-language only in this case report.

## Ethical approval

Ethical approval for this paper was provided by our institution.

## Funding

There is no source of funding found for this paper.

## Author contribution

AAA: Conceptualization, design of the study, acquisition of data, drafting the article, revising it critically for important intellectual content, approval of the version to be submitted.

WTT: Analysis, interpretation of data, drafting the article, revising it critically for important intellectual content, approval of the version to be submitted.

DAA: Conceptualization, analysis, drafting the article, revising it critically for important intellectual content, approval of the version to be submitted.

TSM: Acquisition of data, analysis, revising it critically for important intellectual content, approval of the version to be submitted.

AAA: Acquisition of data, analysis, revising it critically for important intellectual content, approval of the version to be submitted.

WAN: Acquisition of data, analysis, revising it critically for important intellectual content, approval of the version to be submitted.

## Guarantor

Wondale Tsega Tebeje,MD.

^1^Asistant Professor of internal medicine, College of health science Debere Tabor University, Debre Tabor, Ethiopia.

Email = wongotse@gmail.com

Addisu Assfaw Ayen, MD.

^1^Asistant Professor of internal medicine, College of health science Debere Tabor University, Debre Tabor, Ethiopia.

Email =addisuaa1975@gmail.com

## Research registration number

N/A

## Registration of research studies

Not applicable.

## Conflict of interest statement

All authors declare that they have no conflict of interest.
